# Depletion of RIPK1 in hepatocytes exacerbates liver damage in fulminant viral hepatitis

**DOI:** 10.1038/s41419-018-1277-3

**Published:** 2019-01-08

**Authors:** Muhammad Farooq, Aveline Filliol, Mélanie Simoes Eugénio, Claire Piquet-Pellorce, Sarah Dion, Céline Raguenes-Nicol, Aurélien Jan, Marie-Thérèse Dimanche-Boitrel, Jacques Le Seyec, Michel Samson

**Affiliations:** 1 Univ Rennes, Inserm, EHESP, Irset (Institut de recherche en santé, environnement et travail) - UMR_S, 1085 Rennes, France; 2 Department of Clinical Sciences, College of Veterinary and Animal Sciences, Jhang, Pakistan; 3 Present Address: Department of Medicine, Columbia University, New York, NY USA

## Abstract

The protein kinase RIPK1 plays a crucial role at the crossroad of stress-induced signaling pathways that affects cell’s decision to live or die. The present study aimed to define the role of RIPK1 in hepatocytes during fulminant viral hepatitis, a worldwide syndrome mainly observed in hepatitis B virus (HBV) infected patients. Mice deficient for RIPK1, specifically in liver parenchymal cells (*Ripk1*^LPC-KO^) and their wild-type littermates (*Ripk1*^fl/fl^), were challenged by either the murine hepatitis virus type 3 (MHV3) or poly I:C, a synthetic analog of double-stranded RNA mimicking viral pathogen-associated molecular pattern. *Ripk1*^LPC-KO^ mice developed more severe symptoms at early stage of the MHV3-induced fulminant hepatitis. Similarly, administration of poly I:C only triggered increase of systemic transaminases in *Ripk1*^LPC-KO^ mice, reflecting liver damage through induced apoptosis as illustrated by cleaved-caspase 3 labeling of liver tissue sections. Neutralization of TNF-α or prior depletion of macrophages were able to prevent the appearance of apoptosis of hepatocytes in poly I:C-challenged *Ripk1*^LPC-KO^ mice. Moreover, poly I:C never induced direct hepatocyte death in primary culture whatever the murine genotype, while it always stimulated an anti-viral response. Our investigations demonstrated that RIPK1 protects hepatocytes from TNF-α secreted from macrophages during viral induced fulminant hepatitis. These data emphasize the potential worsening risks of an HBV infection in people with polymorphism or homozygous amorphic mutations already described for the *RIPK1* gene.

## Introduction

Fulminant viral hepatitis is a worldwide syndrome, which requires immediate intensive care, and is associated with massive hepatocyte death, hepatic encephalopathy, and multiple organ failure^1^. Even if non-hepatitis viruses, such as the herpes simplex virus (HSV) or the cytomegalovirus (CMV) are occasional etiological factors in immunocompromised patients, most fulminant hepatitis with an identifiable viral cause originates from infection with hepatotropic virus, mainly the hepatitis B virus (HBV) and more rarely the hepatitis A, C, or E viruses (HAV, HCV, HEV)^2,3^. Orthotopic liver transplantation remains the leading life-saving option for patients with fulminant hepatitis^4,5^. Nowadays, mouse models of murine hepatitis virus type 3 (MHV3) infection or polyinosine-polycytidylic acid (poly I:C) injection emerge as effective tools to investigate the underlying mechanisms in pathogenesis and to test novel therapeutic strategies in this disease^6–8^.

Indeed, the more physiologically relevant in vivo model to study fulminant viral hepatitis is MHV3 infection. This coronavirus is a single stranded, positive sense RNA virus. It is highly pathogenic and could cause death in 3–5 days depending on age, route of infection, viral doses, murine strain, and on the immune status of the animal^9^. Different physiopathological issues take place during the course of the disease. In the liver, the cell tropism of MHV3 results in the infection of resident macrophages (Kuppfer cells), liver sinusoidal endothelial cells, hepatic stellate cells, and hepatocytes^10^. Host-pathogen interactions cause release of high levels of inflammatory mediators while repressing the production of immunosuppressive factors^11–14^. Concomitantly, activated macrophages and sinusoidal endothelial cells secrete the prothombinase fibrinogen-like protein 2 (FGL2), initiating fibrin matrix formation. The induced coagulopathy disrupt blood supply leading ultimately to liver necrosis^1^.

Regarding poly I:C, it is a synthetic analog of double-stranded RNA. This pathogen associated molecular pattern (PAMP) interacts with different cellular pattern recognition receptors (PRR), including the Toll like receptor 3 (TLR3), the cytosolic protein kinase RNA-activated (PKR) and the melanoma differentiation-associated protein 5 (MDA5)^15,16^. Upon extracellular double-stranded RNA recognition, TLR3 recruits the cytosolic TIR-domain-containing adapter-inducing interferon-β (TRIF). Through kinases, this adaptor protein induces the nuclear translocation of the interferon regulatory factor 3 (IRF3) which drives the expression of anti-viral type I interferons (IFNs). In another signal transduction cascade, TRIF engages the receptor interacting protein kinase-1 (RIPK1) to promote downstream activation of NF-κB, eliciting the production of pro-inflammatory cytokines, such as TNF-α, IL-6, IL-1β, and chemokines, mainly by Kuppfer cells and natural killer (NK) cells^17,18^. As for Poly I:C, it causes liver damage in mice only if pre-treated with D-galactosamine (D-GalN). This amino sugar specifically blocks the transcription in hepatocytes^19^. Thus, the TNF-α-induced NF-κB pathway cannot lead to the synthesis of the anti-apoptotic proteins. Hepatocytes then die by TNF-α activated apoptosis^8,20^.

TNF-α is a key cytokine involved in both acute and chronic liver diseases, like fulminant hepatic failure, alcohol-induced hepatitis, viral hepatitis, metabolic toxicity, drug-induced liver injury and autoimmune hepatitis. This death factor is recognized by the receptors TNFR1 and TNFR2, but most of its biological activity depends on TNFR1^21^. Downstream of TNFR1, RIPK1 serves as a signaling node to play key roles in regulating cell survival, caspase-dependent apoptosis and RIPK3/mixed lineage domain-like pseudokinase (MLKL)-dependent necroptosis^22^. In previous works, we demonstrated that RIPK1 expressed in liver parenchymal cells provides a protective function during murine hepatitis-induced either directly by TNF-α^23^ or by bacterial pathogen associated molecular patterns (e.g. Lipopolysaccharide or unmethylated CpG oligodeoxynucleotide) which activate Kuppfer cells for TNF-α production^24^. In the present study, we now investigate the unknown role of RIPK1 during the specific circumstances of fulminant viral hepatitis.

## Results

### RIPK1 deficiency sensitized mice to MHV3-induced liver damage

**Fig. 1:**
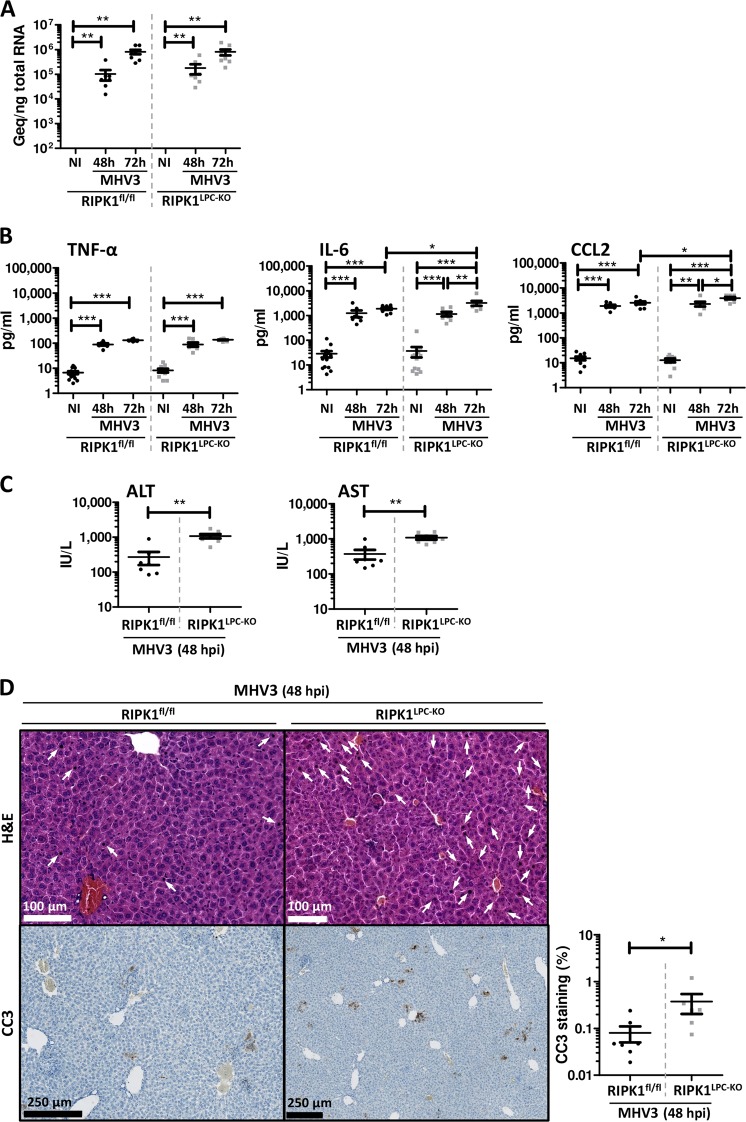
RIPK1 deficiency in liver parenchymal cells partially sensitized mice to viral fulminant hepatitis. *Ripk1*^fl/fl^ and *Ripk1*^LPC-KO^ mice were infected or not (NI) by MHV3. Groups of infected animals were killed 48 or 72 h post inoculation. **a** Quantities of MHV3 genome (Geq: genome equivalent) per ng of total liver RNA. **b** Levels of plasma TNF-α, IL-6, and CCL2. **c** Levels of plasma ALT and AST at 48 h post inoculation. **d** Pictures of liver tissue sections issued from animals killed 48 h post inoculation, stained by H&E (upper panel, white arrows point to pycnotic cells) or analysed by immunohistochemistry for cleaved-caspase-3 (CC3) (lower panel) with signal quantification of cleaved-caspase-3 (low right panel). For all graphs, each black dot and gray square represent a *Ripk1*^fl/fl^ and a *Ripk1*^LPC-KO^ individual, respectively, and errors bars are expressed as means ± SEM

We first investigated the potential functions that RIPK1 could play in liver parenchymal cells during fulminant viral hepatitis by taking advantage of a physiological murine model based on MHV3 infection. A mouse strain deficient for RIPK1 specifically in liver parenchymal cells (*Ripk1*^LPC-KO^) and their controls (*Ripk1*^fl/fl^ littermates) were inoculated with low quantities of viruses. Beforehand, we checked that the introduced genetic modification did not affect the health status of the mouse liver in basal conditions. Indeed, normal transaminase levels were measured in the plasma of these *Ripk1*^LPC-KO^ animals, as for their *Ripk1*^fl/fl^ littermates (87.4 ± 11.1 versus 69.7 ± 13.7 IU/L for ALT, respectively). Similarly, histological observations of their liver did not reveal any anomaly. Animals from both groups were included in an experimental protocol for MHV3-induced fulminant viral hepatitis. The infection levels of each individual were followed by assessing the amount of viral genomes present in their liver at 48 and 72 h post inoculation (hpi) (Fig. 1a). No significant differences were detected between *Ripk1*^LPC-KO^ and *Ripk1*^fl/fl^ mice, showing that RIPK1 deficiency in parenchymal cells of the liver did not alter the MHV3 infection and replication. As pro-inflammatory cytokines play a key role in the development of fulminant viral hepatitis, we investigated their regulations in infected mice. Infection triggered similar cytokine storms in both genotypes as illustrated by the equivalent large quantities found in the bloodstream of *Ripk1*^LPC-KO^ and *Ripk1*^fl/fl^ mice, such as for TNF-α, IL-6, and CCL2 (Fig. 1b). These findings mainly followed the inductions observed at the transcript levels (Supplementary Fig S1B). However, the systemic concentrations of some pro-inflammatory cytokines (IL-6 and CCL2), but not all (TNF-α, IL-1α, and IL-1β), were slightly over-induced in *Ripk1*^LPC-KO^ mice at late stage of infection (72 hpi). Besides, the fulminant hepatitis severity was more pronounced in *Ripk1*^LPC-KO^ mice at 48 hpi. Thus, higher liver damage in *Ripk1*^LPC-KO^ mice were evidenced by elevated plasma AST/ALT transaminases (Fig. 1c). This was confirmed by the histological examination of livers. Accordingly, H&E staining revealed the presence of more pyknosis, a phase preceding cell death, in tissue sections from *Ripk1*^LPC-KO^ compared to those of *RipK1*^fl/fl^ littermates (Fig. 1d, upper panels). Furthermore, *Ripk1*^LPC-KO^ liver sections also revealed higher levels of cleaved caspase-3 (Fig. 1d, lower panels). In fact, as MHV3 continued to replicate in all animals, the hepatitis progressed and all differences observed at 48 hpi disappeared at 72 hpi (Supplementary Fig S2A and S2B).

These findings suggest that RIPK1 plays a partial protective role at early stages of fulminant viral hepatitis.

### Deficiency of RIPK1 in liver parenchymal cells sensitized mice to poly I:C administration

**Fig. 2:**
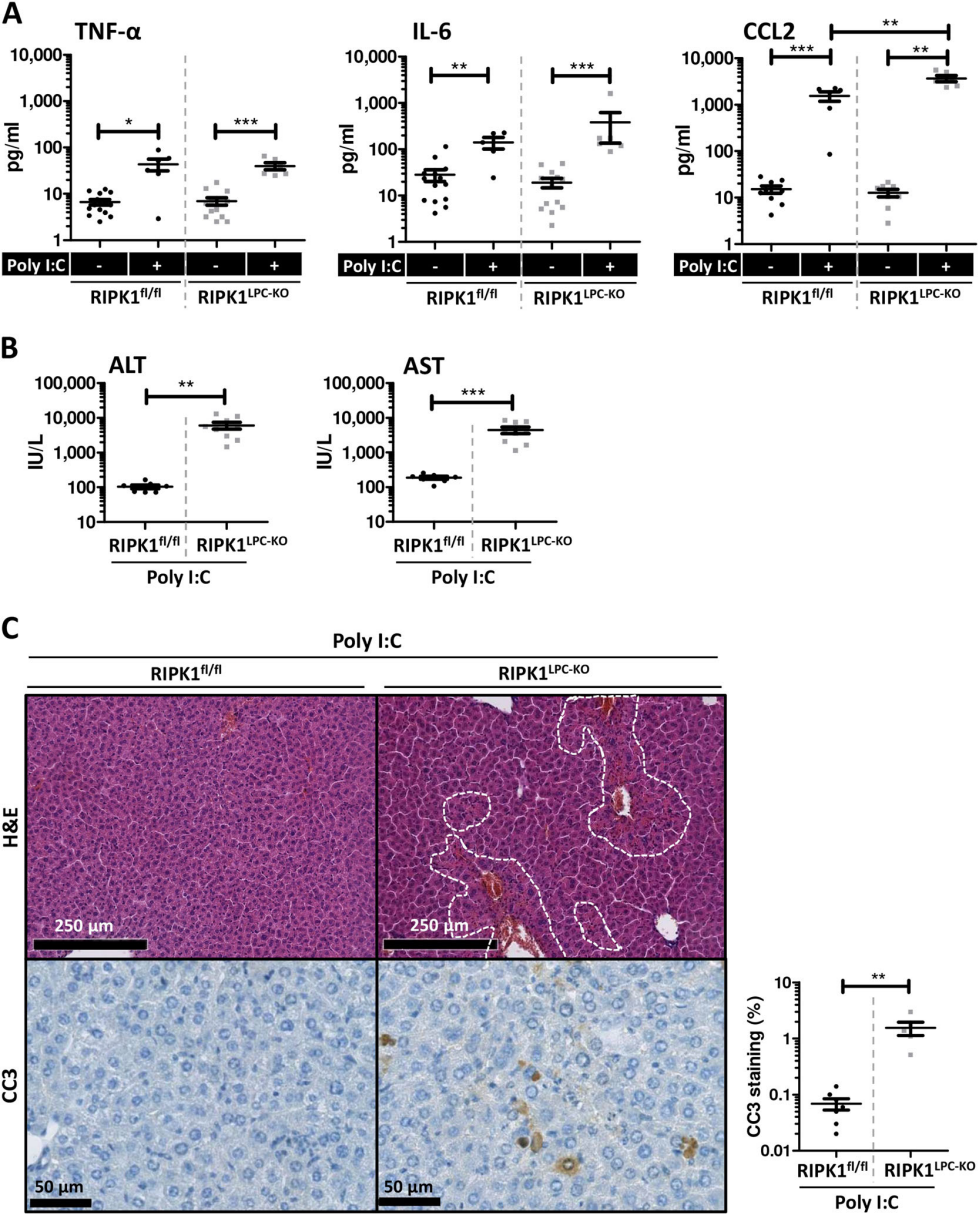
RIPK1 deficiency in liver parenchymal cells sensitized mice to poly I:C-induced hepatitis. PBS or poly I:C were intravenously injected to *Ripk1*^fl/fl^ and *Ripk1*^LPC-KO^ mice 8 h before their euthanasia. **a** Levels of plasma TNF-α, IL-6 and CCL2. **b** Levels of plasma ALT and AST. **c** Pictures of liver tissue sections stained by H&E (upper panel, white dashed lines delimit necrotic areas) or analysed by immunohistochemistry for cleaved-caspase-3 (CC3) (lower panel) with signal quantification of cleaved-caspase-3 (low right panel). For all graphs, each black dot and gray square represent a *Ripk1*^fl/fl^ and a *Ripk1*^LPC-KO^ individual, respectively, and errors bars are expressed as means ± SEM

To pursue investigations on the role of RIPK1 during viral hepatitis, we exposed *Ripk1*^LPC-KO^ and *Ripk1*^fl/fl^ mice to a molecular pattern associated with viral infection, the poly I:C. Adult *Ripk1*^LPC-KO^ and *Ripk1*^fl/fl^ animals were subjected to a unique low dose of poly I:C (1.5 µg/g of body weight) by intravenous injection. The synthetic double-stranded RNA triggered an antiviral response in the liver of both *Ripk1*^LPC-KO^ and *Ripk1*^fl/fl^ mice as demonstrated by the transcriptomic induction of some interferon-inducible antiviral effectors: PKR, the GTP-binding protein Mx1 and the 2’−5’-oligoadenylate synthetase (OAS1c) (Supplementary Fig S3A). To compare the inflammatory response induced in *Ripk1*^LPC-KO^ and *Ripk1*^fl/fl^ mice challenged with poly I:C, we measured the systemic concentrations of a panel of pro-inflammatory cytokines. Whatever the tested murine genotype, administration of synthetic viral nucleic acid pattern resulted in significant increased concentrations of TNF-α, IL-6 and CCL2 (Fig. 2a). Exacerbated inductions were detected in the *Ripk1*^LPC-KO^ strain for the monocyte chemoattractant CCL2. This enhanced upregulation in *Ripk1*^LPC-KO^ mice was found at the transcript level of these 3 cytokines (Supplementary Fig S3B). Regarding plasma transaminases, while both AST and ALT levels remained at physiological concentrations in *RipK1*^fl/fl^ mice 8 h post injection, a massive release of transaminases was observed in the blood of poly I:C-treated *Ripk1*^LPC-KO^ mice (Fig. 2b). On histological examination, liver sections from *Ripk1*^LPC-KO^ revealed more and wider necrotic foci through H&E staining in contrast to their *Ripk1*^fl/fl^ littermates (Fig. 2c, upper panels). Accordingly, some apoptotic cells appeared only in *Ripk1*^LPC-KO^ liver tissues as revealed by cleaved caspase-3 labelling (Fig. 2c, lower panels). These findings suggested that the presence of RIPK1 in hepatocytes helps to protect parenchymal cells from the host immune response triggered by the sensing of viral nucleic acid patterns.

### Neutralization of TNF-α protected *Ripk1*^LPC-KO^ mice from poly I:C-induced liver damage

**Fig. 3:**
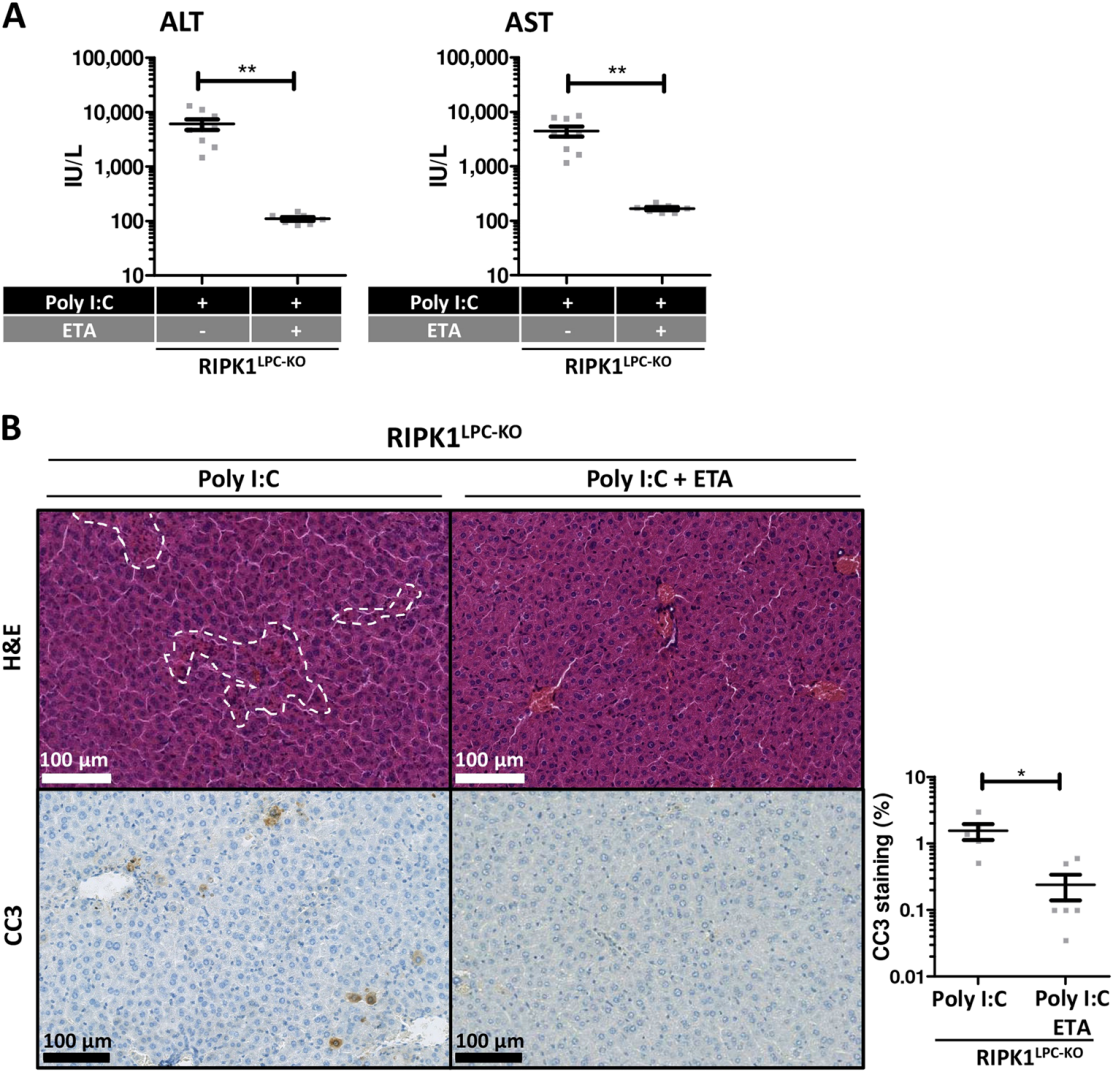
The sensitivity to poly I:C of mice deficient for RIPK1 in their liver parenchymal cells was dependent on the TNF-α cytokine. *Ripk1*^LPC-KO^ mice underwent successively a first intraperitoneal injection of PBS or ETA, then 1 h after a second intravenous injection of poly I:C, and then killed 8 h later. **a** Levels of plasma ALT and AST. **b** Pictures of liver tissue sections stained by H&E (upper panel, white dashed lines delimit necrotic areas) or analysed by immunohistochemistry for cleaved-caspase-3 (CC3) (lower panel) with signal quantification of cleaved-caspase-3 (low right panel). For all graphs, each gray square represents a *Ripk1*^LPC-KO^ individual, and errors bars are expressed as means ± SEM

As highlighted above, an inflammatory response was established in murine individuals exposed to poly I:C. Among released cytokines, blood concentrations of TNF-α were increased by more than five times in *Ripk1*^LPC-KO^ mice as in their *Ripk1*^fl/fl^ littermates (Fig. 2a). In our previous reported studies dealing with non-viral origin hepatitis induced in *Ripk1*^LPC-KO^ mice, death of RIPK1-deficient hepatocytes were directly attributed to high doses of this cytokine^23,24^. To test this mechanistic hypothesis in the hepatitis model induced by a viral pattern, we administered ETA, a decoy receptor of TNF-α, 1 h prior poly I:C injection. Neutralization of TNF-α proved to be sufficient to prevent the occurrence of liver damage in *Ripk1*^LPC-KO^ mice. Indeed, plasma AST/ALT levels remained physiological (Fig. 3a) and little or no necrotic foci or apoptotic cells were distinguished in *Ripk1*^LPC-KO^ liver sections (Fig. 3b). Yet, all the poly I:C injections were successful as shown by PKR, Mx1, and OAS1c mRNA inductions (Supplementary Fig S3C). These data demonstrated that TNF-α was responsible for poly I:C-induced liver damage in *Ripk1*^LPC-KO^ mice.

### Depletion of Kupffer cells reduced poly I:C-induced hepatitis in *Ripk1*^LPC-KO^ mice

**Fig. 4:**
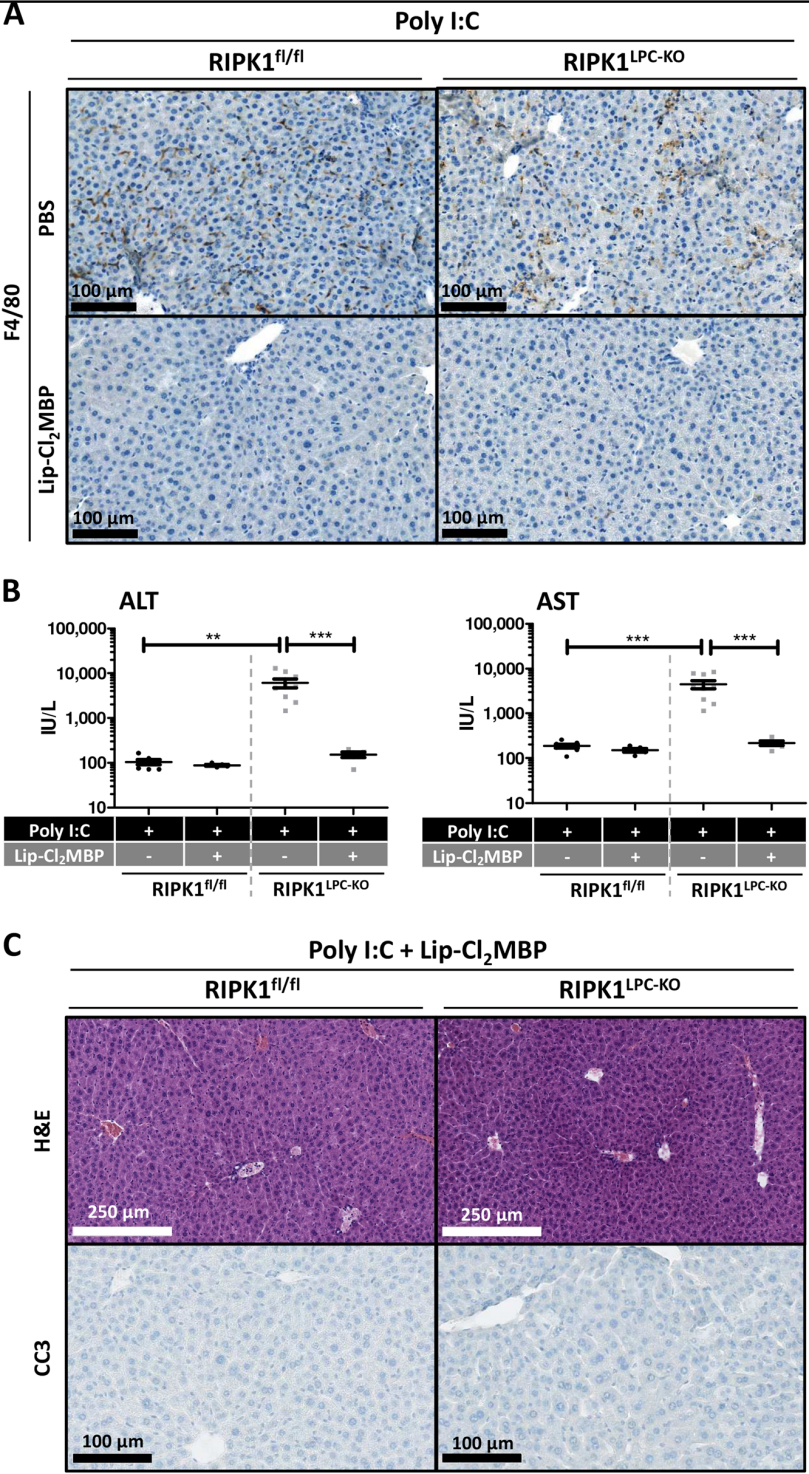
Macrophage depletion protected mice deficient for RIPK1 in their liver parenchymal cells from poly I:C-induced hepatitis. *Ripk1*^fl/fl^ and *Ripk1*^LPC-KO^ mice were successively subjected to two successive intraperitoneal injections of PBS or liposomes-encapsulated Cl_2_MBP at 24 h interval, and then 24 h later, to an intravenous injection of poly I:C and then killed 8 h later. **a** Pictures of liver tissue sections analyzed by immunohistochemistry for F4/80. **b** Levels of plasma ALT and AST. **c** Pictures of liver tissue sections stained by H&E (upper panel) or analysed by immunohistochemistry for cleaved-caspase-3 (CC3) (lower panel). For all graphs, each black dot and gray square represent a *Ripk1*^fl/fl^ and a *Ripk1*^LPC-KO^ individual, respectively, and errors bars are expressed as means ± SEM

At the time of poly I:C exposure, the liver-resident macrophages (Kupffer cells), which express at their membranes the related pattern recognition receptor TLR3, could be one of the sources of the produced TNF-α. This prompted us to specifically deplete the liver of *Ripk1*^LPC-KO^ mice from their Kupffer cells by 2 consecutive intraperitoneal injections of clodronate-loaded liposomes (Lip-Cl_2_MBP) prior to poly I:C administration. F4/80 staining of liver sections confirmed the effectiveness of Kupffer cell depletion (Fig. 4a). Besides, increased expression of hepatic PKR transcripts confirmed that each enrolled mouse was properly challenged by the poly I:C (Supplementary Fig S3D). The *Ripk1*^LPC-KO^ mice lacking Kupffer cells became resistant to poly I:C-induced hepatitis as evidenced by diminished plasma AST/ALT levels (Fig. 4b). Likewise, the livers of these animals showed neither necrotic foci nor cleaved caspase-3 stained areas (Fig. 4c). Macrophages were therefore involved in liver damage observed in *Ripk1*^LPC-KO^ mice challenged with poly I:C.

### Poly I:C was unable to directly induce the death of RIPK1-deficient hepatocytes

**Fig. 5:**
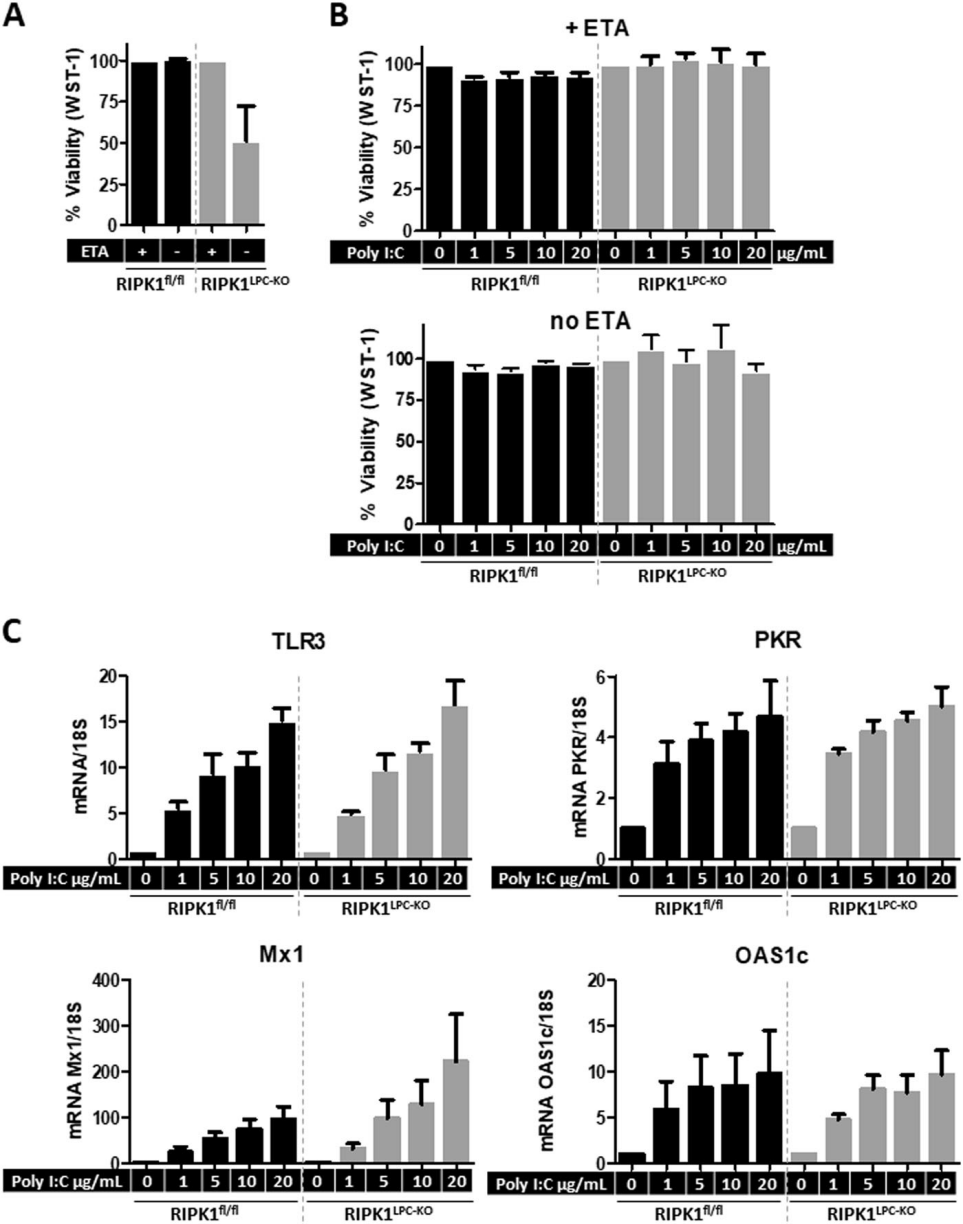
Poly I:C did not induce direct death of RIPK1-deficient primary mouse hepatocytes. Primary cultures of hepatocytes issued from *Ripk1*^fl/fl^ or *Ripk1*^LPC-KO^ mice were maintained 16 h after the seeding period in culture media containing or excluding ETA before cell death analysis by WST-1 based assay. Data are expressed as percentage of signal obtained in basal conditions without poly I:C, in presence or absence of ETA according to the graph. Error bars are expressed as means ± SEM of three-independent experiments. **a** Impact of ETA on cell viability in basal conditions. **b** Impact of increasing concentrations of poly I:C during the 16 h incubation period in presence or absence of ETA on cell viability **c** and on the levels of TLR3, PKR, Mx1 and OAS1c transcripts in cultures maintained in presence of ETA

To investigate the direct impact that poly I:C could have on RIPK1-deficient hepatocytes, primary cultures issued from either *Ripk1*^LPC-KO^ or *Ripk1*^fl/fl^ mice were prepared to be subjected to increasing concentrations of the synthetic double-stranded RNA. In contrast to their WT counterparts, primary cultures of RIPK1-deficient hepatocytes underwent substantial spontaneous death, as previously reported^23–27^ (Fig. 5a). This death occurred by apoptosis since it was completely prevented by a pan-caspase inhibitor (z-VAD-fmk) (data not shown)^27^. Death in RIPK1-deficient hepatocyte primary cultures could be largely avoided by adding ETA to culture medium (Fig. 5a)^27^. This demonstrated that their death originated from sensing autocrine TNF-α. The limited remaining observed death in ETA-treated RIPK1-deficient hepatocyte primary cultures could probably be attributed in part to cells already engaged in TNF-α signaling before ETA addition. When ETA was added to the culture medium to avoid spontaneous death of RIPK1-deficient hepatocytes, poly I:C, even at high doses (20 µg/mL), never induced the death of hepatocytes regardless of their genotype (Fig. 5b, upper panel). Experiments conducted in parallel on same batches of primary hepatocytes, but in absence of ETA, showed that addition of poly I:C, whatever the tested dose, did not cause any additional death (Fig. 5b, lower panel). Thus, in these conditions, death of RIPK1-deficient hepatocytes induced by endogenous TNF-α was not potentiated by poly I:C. For all these experiments, the efficacy of poly I:C signaling on primary mouse hepatocytes was checked by measuring the transcript levels of genes typically induced (TLR3, PKR, Mx1, and OAS1c). Dose-dependent upregulations were consistently observed for all tested genes, both in control and deficient hepatocytes (Fig. 5c). These findings further strengthened our notion of involvement of TNF-α, secreted by Kupffer cells, in poly I:C-induced liver damage in *Ripk1*^LPC-KO^ mice.

## Discussion

Hepatocytes express the cytosolic kinase RIPK1 which has been described to be at the crossroad of signal transduction pathways that regulate cell fate. Indeed, RIPK1 helps to promote cell survival or switches cell to death by caspase-dependent apoptosis or by MLKL-dependent necroptosis^18^. During fulminant viral hepatitis, a cytokine burst arises, resulting in an enrichment of the hepatic microenvironment in death factors such as TNF-α, Fas ligand (FasL) or TRAIL. Once bound to their cognate receptors expressed on hepatocyte surface, the induced intracellular signal must involve RIPK1 which should impact the disease outcome, systematically characterized by massive hepatocyte death. To investigate the role of RIPK1 in fulminant viral hepatitis, mice with either normal hepatocytes (*Ripk1*^fl/fl^) or defective hepatocytes for this kinase (*Ripk1*^LPC-KO^) were inoculated with the MHV3. Whatever the murine genotype, all animals were equally susceptible to MHV3 infection as illustrated by the similar amounts of replicative genomes detected in their livers at identical time points. Most of tested clinical and biochemical parameters of fulminant hepatitis were similar between both infected mice cohorts, especially at the final stage of the infection. In particular, no difference was distinguished between the two groups of animals for parameters that influence the induction of the prothrombinase FGL2 expression. This pro-coagulant factor, which is at the origin of hemostasis alterations and coagulation, takes part in the pathogenicity of the MHV3 infection. Indeed, antibodies against FGL2^28^ or its genetic invalidation^29^ have been described to significantly decrease the severity of MHV3-induced hepatitis. Its production is known to be triggered by a viral factor (nucleocapsid)^30^ and by both cytokines TNF-α and IFNγ^31^. The lack of RIPK1 in liver parenchymal cells never altered levels of viral replication and that of the induced plasma concentrations of the above-mentioned cytokines (Fig. 1a, b and Supplementary Fig S1C), leading to similar FGL2 mRNA inductions (Supplementary Fig S1B). However, even if all animals succumbed at day 4 post-inoculation, *Ripk1*^LPC-KO^ mice showed more severe signs of liver damage (plasma transaminases, apoptotic hepatocytes) at an earlier stage of infection. Although the host defense system is insufficient to protect C57BL/6 mice from fatal MHV3 fulminant hepatitis, RIPK1 appeared to partly contribute to some resistance underlying the protective function played by RIPK1 in hepatocytes. The observed partial worsening of symptoms in MHV3-infected *Ripk1*^LPC-KO^ mice most probably resulted at least from their described increased sensitivity for both TNF-α and FasL^23,25,26^. Indeed, these death factors directly contribute to the death of hepatocytes and thus to pathogenesis in MHV3 fulminant hepatitis. Accordingly, significant resistance has been described for animals deficient for TNF-α or TNFR1 when infected by MHV3^32,33^. Similarly, Kuppfer cells displays more FasL at their surface in a MHV3-infected mouse, while hepatocytes also overexpress the cognate receptor (Fas). In addition, ex vivo experiments showed that a neutralizing antibody directed against FasL prevents the death of infected hepatocytes by liver natural killer (NK) cells^34^.

Both mouse strains were also enrolled in a surrogate model based on injection of synthetic double-stranded RNA (poly I:C) mimicking replicative forms of viral genomes. Usually, to elicit a TLR3-regulated fulminant viral hepatitis, poly I:C should be used in combination with D-galactosamine (D-GalN)^8^, a liver-specific transcriptional inhibitor^19^. However, a simple injection of poly I:C was sufficient to induce hepatitis in *Ripk1*^LPC-KO^ mice, without affecting the control littermates (*Ripk1*^fl-fl^). Even if poly I:C stimulated an antiviral innate response in primary hepatocytes issued either from *Ripk1*^fl/fl^ or *Ripk1*^LPC-KO^ mice, it never induced direct cell death or sensitized hepatocytes to TNF-α. In contrast, it has been demonstrated that in some RIPK1-deficient cell types, such as mouse embryonic fibroblasts or primary human fibroblasts, poly I:C directly induced RIPK3-dependent necroptosis^35–37^. This discrepancy could probably be explained by the limited expression of RIPK3 in hepatocytes^38,39^. Poly I:C-treated animals with RIPK1-deficient hepatocytes developed the usual symptoms of acute liver failure accompanied by an enhanced systemic release of TNF-α, which was responsible for hepatocyte death. Indeed, neutralization of TNF-α by a decoy receptor (ETA) was sufficient to prevent any clinical manifestations in vivo and to protect primary RIPK1-deficient hepatocytes from apoptosis induced by autocrine TNF-α. This pro-inflammatory cytokine turned into a direct death-inducer for hepatocytes when deficient in RIPK1. Thus, the presence of this kinase in hepatocytes protects them from death when TNF-α appears in their microenvironment, whatever the origin of the inflammatory context. Here TNF-α, which emerged from a response to a viral pathogen associated molecular pattern (PAMP) recognized by the TLR3, was most probably produced by Kupffer cells as their previous chemical depletion prevented the development of hepatitis in poly I:C challenged *Ripk1*^LPC-KO^ mice. The protective property of RIPK1 against TNF-α was also observed for immune responses originating from bacterial PAMPs detected by TLR4 and TLR9 (lipopolysaccharides and unmethylated CpG oligodeoxynucleotide, respectively)^24^ and in an acute hepatic autoimmune model induced by concanavalin A injection^23^. RIPK1 therefore appears to be an important part of the hepatocyte protection system, especially when the liver undergoes different types of insults, highlighting the potential vulnerability of patients who may harbor already described genetic polymorphism altering the hepatic disease outcome^40^ or homozygous amorphic mutations^36^.

## Materials and methods

### Ethics statement

Animal studies were reviewed and approved by the “Comité d’Ethique en Expérimentation Animale” (C2EA – 07) under the French Ministry of Higher Education and Research (permission#: 10460–2017070300162663 v3). The study was carried out in strict accordance with the recommendations in the Guide for the Care and Use of Laboratory Animals, EEC Council Directive 2010/63/EU.

### Animals, virus, and treatment protocols

For MHV3 experiments, animals were maintained in individually ventilated cages (Forma Scientific, 1 Marietta, OH) in the BSL3 local animal facility. *Ripk1*^LPC-KO^ C57BL/6 mice have been already described in previous works^23^. For each experiment, these genetically modified mice were systematically compared to their WT *Ripk1*^fl/fl^ littermates. Homogeneous groups of male and female mice at 8–20 weeks of age were used for each experiment. Genotyping was performed by conventional PCR for Alfp-Cre gene from DNA extracted from tail samples of mice^24^.

For in vivo viral inoculation, the pathogenic L2-MHV3 strain were injected by intraperitoneal route at 10^3^ 50% tissue culture infective dose (TCID_50_) per animal as described previously^41^. Mice were followed up twice a day for weight loss (Supplementary Fig S1A). At 48 h and 72 h post infection, mice were killed by cerebral dislocation before liver and blood sampling. Other mice were followed up for 4 days for survival monitoring.

Poly I:C (Invivogen) diluted in PBS was intravenously administered at a dose of 1.5 mg/kg body weight. Etanercept (ETA, Pfizer) was injected via intraperitoneal route at a dose of 10 mg/kg body weight (10 μL/g body weight) 1 h prior poly I:C challenge. When indicated, macrophages were depleted by liposomes-encapsulated dichloromethylene bis-phosphonate (Cl_2_MBP) i.p injections as described previously^24,42^. Briefly, Cl_2_MBP was administered twice (with 24 h interval between injections) in mice. The first and second injections were respectively given at the rate of 10 and then 5 μl/g body weight. Blood was collected before injecting poly I:C, which was injected 48 h after first injection of liposomes-encapsulated Cl_2_MBP and killed 8 h post poly I:C injection.

### Histopathological and biochemical studies

Mouse liver fragments were fixed in 4% paraformaldehyde and embedded in paraffin for immunohistochemistry. For histopathology, hematoxylin and eosin (H&E) staining of liver tissues was carried out to investigate the liver injury. Plasma alanine (ALT) and aspartate (AST) transaminases were measured according to the IFCC primary reference procedures using Olympus AU2700 Auto- analyser^®^ (Olympus Optical, Tokyo, Japan).

### Immunolocalization in liver tissues

For immunolocalization in liver tissues, paraffin-embedded mouse liver sections (5 µm) were dried for 1 h at 58 °C, followed by antigen retrieval and incubation with primary antibody (anti-cleaved caspase-3, Cell Signaling, #9661; anti-F4/80, eBioscience, #12–4801–80) in a Ventana automated instrument (Ventana Medical Systems, USA). Revelation of primary antibody was carried out using horse-radish peroxidase-conjugated secondary antibody (Dako, USA). All paraffin-embedded mouse liver sections were scanned with a digital slide scanner (Hamamatsu, Nanozoomer 2.0-RS) and files were analysed with the NDP viewer software.

### RNA isolation and RT-qPCR

Total RNA was extracted from mice liver tissues and from primary hepatocytes using the NucleoSpin^®^ RNA kit (Macherey-Nagel, #740955). First-strand cDNA was synthesized using the High-Capacity cDNA Reverse Transcription Kit (Applied Biosystems, #4368813, Foster City, CA, USA). Real-time quantitative PCR was performed using the double-strand specific SYBR^®^ Green system (Applied Biosystems, #4367659) on CFX384 Touch^TM^ Real-Time PCR Detection System (Biorad). Each measurement was performed in triplicate. The relative gene expression was normalized against the 18S gene expression. The PBS-treated mice served as reference for mRNA expression (control mRNA level was arbitrarily set at 1). For the absolute quantification of the MHV3 genome by qPCR, a plasmid (pBAC-JHMV^IA^)^43^, containing the entire genome of the neurotropic JHM strain of MHV, was used for calibration. The primer sequences are all depicted in Table S1 in supporting information.

### Plasma cytokine immunoassay by flow cytometry

Murine cytokines were quantified by bead-based immunoassays according to the manufacturer protocol, using a filter plate and a vacuum filtration system for washing steps (LEGENDplex^TM^ Mouse Inflammation Panel kit, BioLegend). Samples were analyzed on a LSR Fortessa cytometer (BD Biosciences).

### Primary hepatocyte isolation and culture

Murine hepatocytes were isolated and purified from adult *Ripk1*^*fl/fl*^ or *Ripk1*^*LPC-KO*^ mice as described previously^25^ with minor modifications. The perfused liver was first washed with solution I (8 g/l NaCl, 0.2 g/l KCl, 0.1 g/l Na_2_HPO_4_.12 H_2_O, 2.38 g/l HEPES, pH 7.6 and 0.5 mM EGTA) at a 10 mL/min flow rate for 8–10 min. Then, the perfusion solution I without EGTA was supplemented with 5 mM CaCl_2_.2H_2_O and 0.01% collagenase type 4 (Worthington Biochemical Corporation, Serlabo Technologies, Entraigues, France) at a 7 mL/min flow rate for 5–7 min. After the completed isolation process, hepatocytes were seeded at a density of 6 × 10^4^ cells/cm^2^ in 96-well plates and 24-well plates, previously coated with collagen type I (BD Biosciences), in Williams’ E medium supplemented with 10% (vol/vol) fetal calf serum, 2 mM glutamine, 10 IU/mL penicillin, 10 μg/mL streptomycin and 5 μg/mL insulin. Around 4 h post plating, cells were washed twice with PBS before their stimulation with poly I:C in a similar medium that the seeding supplemented Williams’ E medium in which fetal calf serum was replaced by 1 mg/mL bovine serum albumin with or without 1 μg/mL of ETA (Pfizer). Cell viability was evaluated after a treatment period of 16 h with the Cell Proliferation Reagent WST-1 (Roche), according to the manufacturer’s instructions. Cells in 24 wells were processed for RT-qPCR analysis.

### Statistical analysis

Data were expressed as means ± SEM for all mice treated similarly. Mean differences between experimental groups were assessed using the non-parametric Mann–Whitney *U*-test. Statistical analysis for the in vitro experiments were performed using the unpaired Student’s *t*-test. All statistical analysis was achieved with the GraphPad Prism5 software. Significance is shown as follows: *P < 0.05, **P *<* 0.01, ***P *<* 0.001.

## Supplementary information

Figure S1

Figure S2

Figure S3

Supplementary figure legends

TableS1
