# Supplementary figures and images for: Depletion of RIPK1 in hepatocytes exacerbates liver damage in fulminant viral hepatitis
Source: Cell Death Dis. 2019 Jan 8;10(1):12. doi: 10.1038/s41419-018-1277-3 (PMC6325114; doi:10.1038/s41419-018-1277-3)

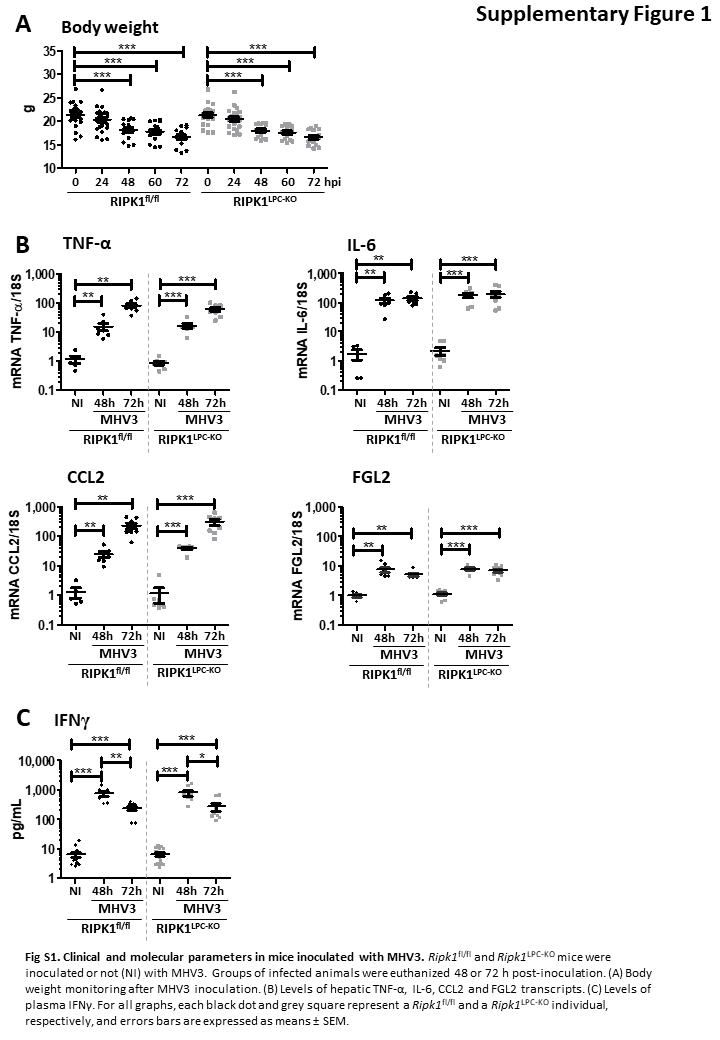

Supplement: Supplementary file 1 — Figure S1 [file 41419_2018_1277_MOESM1_ESM.tif]

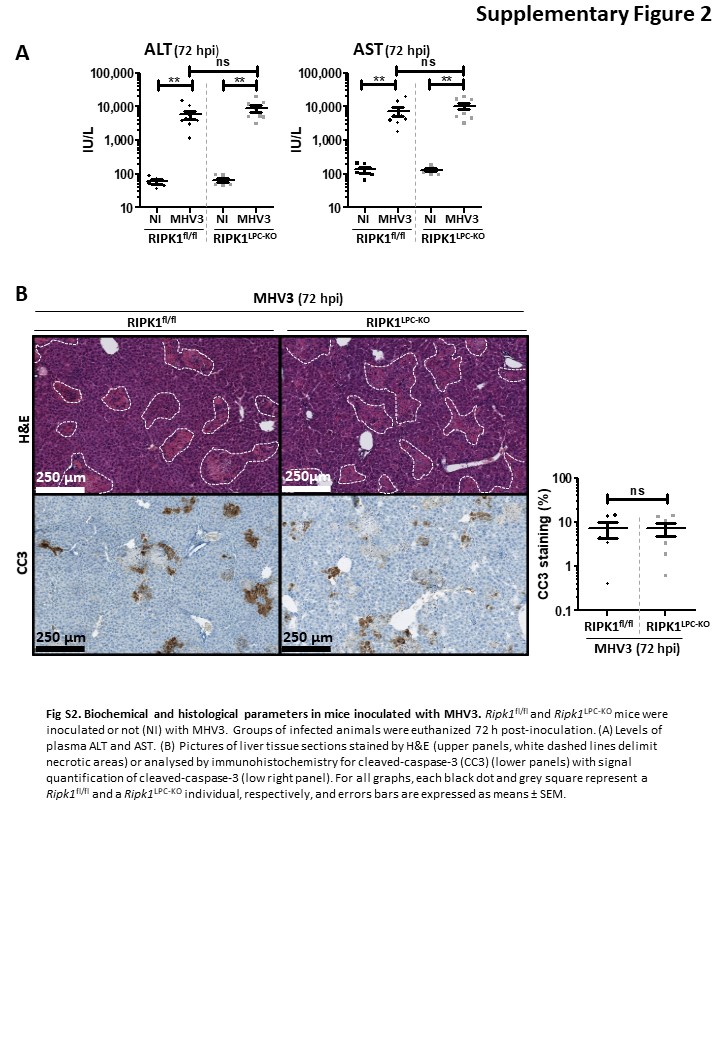

Supplement: Supplementary file 2 — Figure S2 [file 41419_2018_1277_MOESM2_ESM.jpg]

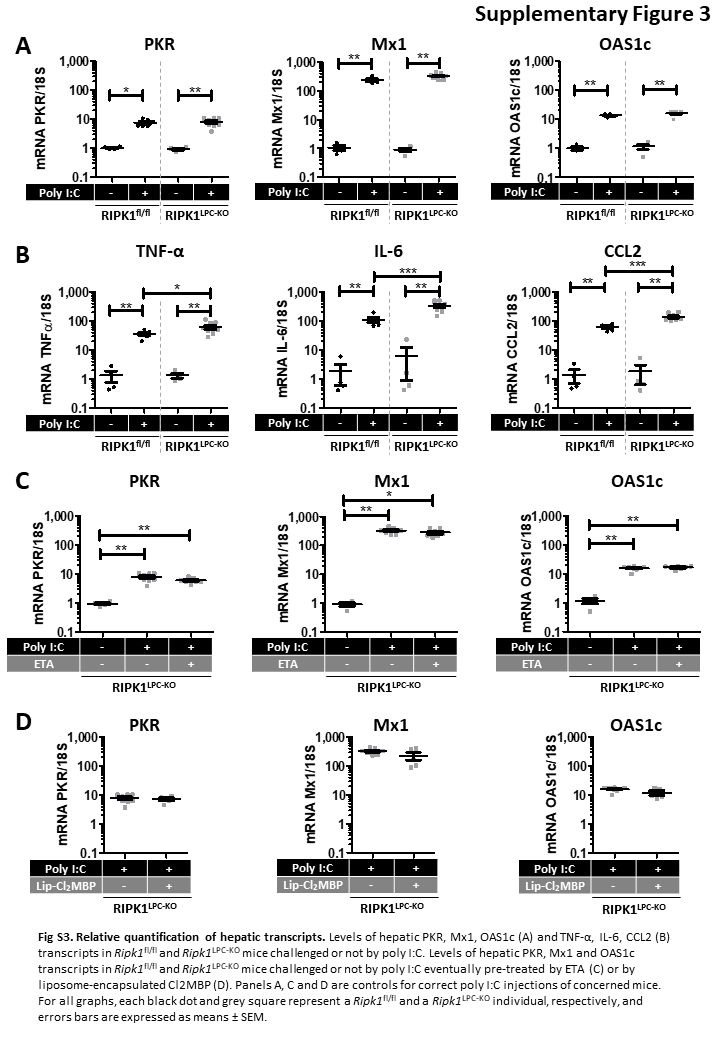

Supplement: Supplementary file 3 — Figure S3 [file 41419_2018_1277_MOESM3_ESM.tif]
