# Supplementary material for: Depletion of RIPK1 in hepatocytes exacerbates liver damage in fulminant viral hepatitis
Source: Cell Death Dis. 2019 Jan 8;10(1):12. doi: 10.1038/s41419-018-1277-3 (PMC6325114; doi:10.1038/s41419-018-1277-3)
Supplement: Supplementary file 4 — Supplementary figure legends [file 41419_2018_1277_MOESM4_ESM.docx]

**Supplementary figure legends**

**Fig S1. Clinical and molecular parameters in mice inoculated with** **MHV3.** *Ripk1*^fl/fl^ and *Ripk1*^LPC-KO^ mice were inoculated or not (NI) with MHV3. Groups of infected animals were euthanized 48 or 72 h post-inoculation. (A) Body weight monitoring after MHV3 inoculation. (B) Levels of hepatic TNF-α, IL-6, CCL2 and FGL2 transcripts. (C) Levels of plasma IFNγ. For all graphs, each black dot and grey square represent a *Ripk1*^fl/fl^ and a *Ripk1*^LPC-KO^ individual, respectively, and errors bars are expressed as means ± SEM.

**Fig S2. Biochemical and histological parameters in mice inoculated with** **MHV3.** *Ripk1*^fl/fl^ and *Ripk1*^LPC-KO^ mice were inoculated or not (NI) with MHV3. Groups of infected animals were euthanized 72 h post-inoculation. (A) Levels of plasma ALT and AST. (B) Pictures of liver tissue sections stained by H&E (upper panel, white dashed lines delimit necrotic areas) or analysed by immunohistochemistry for cleaved-caspase-3 (CC3) (lower panel) with signal quantification of cleaved-caspase-3 (low right panel). For all graphs, each black dot and grey square represent a *Ripk1*^fl/fl^ and a *Ripk1*^LPC-KO^ individual, respectively, and errors bars are expressed as means ± SEM.

**Fig S3. Relative quantification of hepatic transcripts.** Levels of hepatic PKR, Mx1, OAS1c (A) and TNF-α, IL-6, CCL2 (B) transcripts in *Ripk1*^fl/fl^ and *Ripk1*^LPC-KO^ mice challenged or not by poly I:C. Levels of hepatic PKR, Mx1 and OAS1c transcripts in *Ripk1*^fl/fl^ and *Ripk1*^LPC-KO^ mice challenged or not by poly I:C eventually pre-treated by ETA (C) or by liposome-encapsulated Cl_2_MBP (D). Panels A, C and D are controls for correct poly I:C injections of concerned mice. For all graphs, each black dot and grey square represent a *Ripk1*^fl/fl^ and a *Ripk1*^LPC-KO^ individual, respectively, and errors bars are expressed as means ± SEM.
