# Supplementary material for: Depletion of RIPK1 in hepatocytes exacerbates liver damage in fulminant viral hepatitis
Source: Cell Death Dis. 2019 Jan 8;10(1):12. doi: 10.1038/s41419-018-1277-3 (PMC6325114; doi:10.1038/s41419-018-1277-3)
Supplement: Supplementary file 5 — TableS1 [file 41419_2018_1277_MOESM5_ESM.doc]

Table S1. Sequences of primers used for qPCR

| **Gene** | **Forward primer** (5’-3’) | **Reverse**  **primer** (5’-3’) |
| --- | --- | --- |
| *Tnf* | TAGCTCCCAGAAAAGCAAGC | TTTTCTGGAGGGAGATGTGG |
| *Il6* | CGATGATGCACTTGCAGA | CTCTGAAGGACTCTGGCTTTG |
| *Tlr3* | CACGAACCTGACAGAACTCG | CACTTCGCAACGCAAGG |
| *Eif2ak2* (PKR) | GATGGAAAATCCCGAACAAGGAG | AGGCCCAAAGCAAAGATGTCCAC |
| *Mx1* | CCTGGAGGAGCAGAGTGACAC | GGTTAATCGGAGAATTTGGCAA |
| *Oas1c* | TAGAATCCATGGCTCCTGCT | AGAATCAGTGCACCACGATG |
| *Rn18s* | TTGGCAAATGCTTTCGCTC | CGCCGCTAGAGGTGAAATTC |
| *Alfp-Cre* | GCCTGCATTACCGGTCGATGCAACGA | GTGGCAGATGGCGCGGCAACACCATT |
| MHV3 Capsid | TGGAAGGTCTGCACCTGCTA | TTTGGCCCACGGGATTG |
